# Case report: VA-ECMO for fulminant myocarditis in an infant with acute COVID-19

**DOI:** 10.3389/fped.2023.1180534

**Published:** 2023-06-27

**Authors:** Hao-Ting Hsu, Ni-Chun Kuo, Po-Yen Chen, Sheng-Ling Jan

**Affiliations:** ^1^ Department of Pediatrics, Children's Medical Center, Taichung Veterans General Hospital, Taichung, Taiwan; ^2^ Department of Post-Baccalaureate Medicine, College of Medicine, National Chung Hsing University, Taichung, Taiwan; ^3^ Department of Pediatrics, School of Medicine, National Yang-Ming University, Taipei, Taiwan; ^4^ Department of Pediatrics, School of Medicine, Kaohsiung Medical University, Kaohsiung, Taiwan

**Keywords:** VA-ECMO, coronavirus disease 2019, COVID-19, fulminant myocarditis, pediatric, myocarditis

## Abstract

Fulminant myocarditis in children was rare during the coronavirus disease 2019 pandemic, but it had the potential for high morbidity and mortality. We describe the clinical course of a previously healthy 9-month-old young male infant who rapidly deteriorated into cardiogenic shock due to coronavirus disease 2019-related fulminant myocarditis. He developed severe heart failure and multiple organ dysfunction syndrome that were treated promptly with central venoarterial extracorporeal membrane oxygenation and continuous venovenous hemofiltration. He made a good recovery without significant morbidity.

## Introduction

The coronavirus disease 2019 (COVID-19) pandemic was caused by severe acute respiratory syndrome coronavirus 2 (SARS-CoV-2), which spread worldwide at a rapid pace, resulting in significant mortality and morbidity. As the variants of SARS-CoV-2 subsequently appeared with variable clinical and epidemiological traits, COVID-19 has been spreading rapidly in Taiwan since April, 2022. Although acute SARS-CoV-2 infection is often asymptomatic or mild in children (1), some cases of severe infection within the pediatric population have been documented, including acute respiratory distress syndrome (ARDS) (2), encephalitis (3), and acute myocarditis (4–15), but life-threatening complications, such as fulminant myocarditis, are rare. Here, we report a case of acute COVID-19 complicated with fulminant myocarditis in a 9-month-old infant. He required venoarterial extracorporeal membrane oxygenation (VA-ECMO) support due to severe cardiogenic shock, and fully recovered with an excellent outcome.

## Case report

The patient was a 9-month-old previously healthy male infant who presented to the emergency department on 11 July, 2022 with one day of shortness of breath. Initially, he became irritable one day ago, followed by vomiting once, poor appetite, and decreased urine output. A diagnosis of acute gastroenteritis was made at a local clinic. However, fever and dyspnea developed the next day. He was thus brought to the pediatric emergency department of Taichung Veterans General Hospital (TCVGH), a tertiary medical center in central Taiwan. Upon examination, he was found to be febrile with a body temperature of 38.2°C and was tachycardic with a heart rate of 180 bpm. He had poor eye contact and activity, had cyanosis of the lips, and bilateral wheezing breath sounds, accompanied with subcostal retractions. His respiratory distress deteriorated despite epinephrine inhalation provided under the impression of acute bronchiolitis. Thus, he was intubated.

Blood tests revealed a remarkable elevation in lactate (124.5 mg/dl, normal 3–12 mg/dl), leukocytosis with a white blood cell count of 17,050 cells/μl, anemia with hemoglobin of 10.5 g/dl, thrombocytosis with a platelet count of 482 × 10^3 ^cells/μl, mild elevation of aspartate aminotransferase (AST) level (66 U/L, normal 8–38 U/L), elevated cardiac troponin I (1.06 ng/ml, normal ≦0.16 ng/ml) and N-terminal pro-B-type natriuretic peptide (NT-proBNP) (>35,000 pg/ml, normal <400 pg/ml), elevated creatine kinase-myocardial band (CK-MB) level (21 U/L, normal <16 U/L), and normal creatine kinase (CK) level (153 U/L, normal 10–160 U/L). Venous blood gas (VBG) analysis showed severe metabolic acidosis with pH of 7.126, bicarbonate (HCO_3_^−^) of 10.9 mmol/L, and base excess (BE) of −17.2. There was no increase in C-reactive protein (CRP) level (0.036, normal <0.3 mg/dl). The patient had close contact with his father who was a confirmed COVID-19 case. Rapid real-time polymerase chain reaction (RT-PCR) test for SARS-CoV-2 from a nasopharyngeal swab showed a positive result with a cycle threshold (Ct) value of 20.

The patient later became hypotensive (63/43 mmHg) and bradycardic (59 bpm), and thus fluid challenge and cardiopulmonary resuscitation (CPR) were provided, as well as administration of intravenous (IV) epinephrine and sodium bicarbonate. Tachycardia developed after CPR, with a heart rate up to 203 bpm. Electrocardiogram showed sinus tachycardia. Amiodarone and dopamine continuous infusion were given because of persistent tachycardia and hypotension. The emergency medicine physician performed point-of-care ultrasonography, which revealed a dilated heart with poor wall motions. The left ventricular ejection fraction (LVEF) was 20%.

According to the clinical manifestations and detection of SARS-CoV-2 by RT-PCR, the patient's condition was consistent with cardiogenic shock complicated by acute SARS-CoV-2 infection-related fulminant myocarditis. He was then admitted to the negative pressure room of the pediatric intensive care unit on 12 July, 2022.

After hospitalization, central and peripheral access lines were placed. The patient was initially managed by positive pressure mechanical ventilation and administration of milrinone, norepinephrine, dobutamine, epinephrine and dopamine, sequentially or alternately as indicated according to clinical conditions. The patient also received empiric meropenem due to concern of superimposed bacterial infection. Remdesivir was initiated with a dose of 10 mg/kg for one day, followed by 5 mg/kg for 2 days. An intravenous immunoglobulin (IVIG) dose of 1 g/kg was also given once. CPR was performed again due to bradycardic and hypotensive episodes. Bedside echocardiogram revealed deteriorated LVEF of 19%. Advanced cardiovascular support was initiated with VA-ECMO on the same day of admission. Continuous venovenous hemofiltration (CVVH) was also applied for acute renal failure and increased clearance of cytokines.

**Figure 1:**
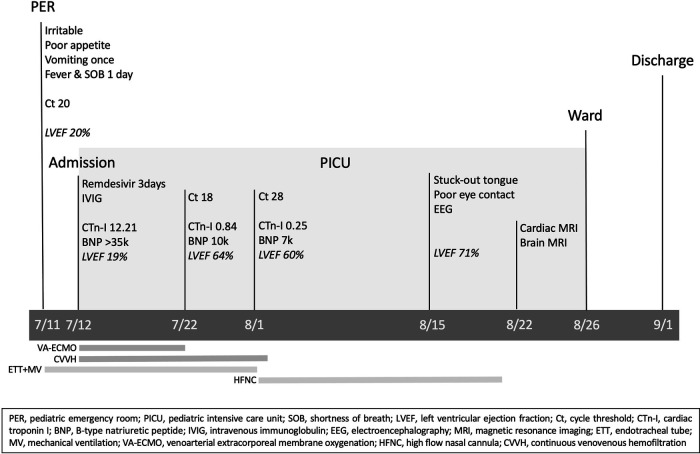
Case timeline.

Under the support of VA-ECMO and CVVH, the patient's condition gradually stabilized and VA-ECMO was weaned off after 10 days. The blood tests conducted one week after the removal of ECMO showed significant improvements in CK, CK-MB, cardiac troponin I, and NT-proBNP. Follow-up of LVEF showed significant improvement from 19% to 60%. The subsequent data are shown in detail in Table 1. The renal replacement therapy with CVVH was applied for a total of 22 days. Extubation was performed after 21 days of mechanical ventilation and a high-flow nasal cannula (HFNC) oxygen therapy was provided for another 20 days before the patient could tolerate ambient air. On the 35th day of hospitalization, the patient stuck his tongue out and had relatively poor eye contact. Electroencephalography was indicative of diffuse cortical dysfunction. Brain magnetic resonance imaging (MRI) showed cortical T2/FLAIR hyperintensity with corresponding T1 hyperintensity and post-contrast gyriform enhancement at bilateral cerebral cortices, compatible with previous ischemic encephalopathy. Cardiac MRI was done on the 40th day of hospitalization, which showed mildly increased T2 signal intensity and late enhancement in the myocardium of the anterior wall of the right atrium and right ventricle, especially over the junction of the right atrium and ventricle, compatible with myocarditis. He recovered almost completely, with only trivial neurological sequelae, and was discharged with outpatient follow-up and rehabilitation. The whole course of this case is demonstrated with a timeline in Figure 1.

**Table 1:** The laboratory tests of the patient during hospitalization.

	Admission	ECMO day 1	During ECMO peak level	Off ECMO 1 week	Normal range
WBC, /μl	17,050	30,040	30,040	8,890	5,300–12,000
Hemoglobin, g/dl	10.5	10.1	5.5	10.9	11.6–13.7
Platelet count, ×10^3^/μl	482	333	39	121	150–400
AST, U/L	66	7,966	7,966	128	8–38
ALT, U/L	23	2,430	2,430	3	10–50
Bilirubin (T/D), mg/dl	–	4.44/1.81	13.37/5.72	8.6/4.4	<1.2/<0.2
Creatinine, mg/dl	0.51	1.11	1.33	0.87	0.5–1.2
C-reactive protein, mg/L	0.036	Nil	Nil	3.193	<0.3
CK, U/L	153	3,631	8,372	48	24–195
CK-MB, U/L	21	97	105	15	<25
CTn-I, ng/ml	1.06	12.21	12.21	0.25	<0.16
NT-proBNP, pg/ml	–	>35,000	>35,000	7,297	<125
LVEF (%)	20%	19%	19%	60%	56%–78%

WBC, white blood cell; AST, aspartate aminotransferase; ALT, alanine transaminase; CK, creatine kinase; CK-MB, creatine kinase isoenzyme; CTn-I, cardiac troponin I; NT-proBNP, N-terminal pro–B-type natriuretic peptide; LVEF, left ventricular ejection fraction.

## Discussion

Acute myocarditis is a known complication associated with viral illnesses and is the result of interactions between the virus and the host's immune system (16). Infection with viral pathogens, such as influenza and parvovirus B-19, has been widely described as the most common etiology of acute myocarditis, but less is known about cardiac complications of SARS-CoV-2 infection. While myocardial problems associated with SARS-CoV-2 have been well reported in adults, few cases of pediatric COVID-19-related acute myocarditis have been reported to date (4–15). We collected twelve such case reports, as shown in Table 2. In these patients, there was no prior history of coronary artery disease, atrial fibrillation, or heart failure. The average onset day was three days. Median age was 9.5 years old. Patients with acute myocarditis from COVID-19 can present initially with nonspecific symptoms, such as fever, dyspnea, or chest pain, making the diagnosis difficult to recognize within the early stages of the disease (14). In the case series of twelve cases, almost all cases had febrile episodes. Fatigue and vomiting were the most common initial symptoms. All cases had elevated cardiac troponin. Five cases presented with ST elevation by electrocardiogram. Three cases showed cardiac conduction disorders. Various levels of decreased cardiac systolic function were disclosed by a decline of ejection fraction or fractional shortening via echocardiography. Four cases required CPR. Five cases required ECMO cannulation. No known mortality was reported in these cases.

**Table 2:** Case reports of children with acute myocarditis after COVID-19.

Reference	Age	Sex	Onset	Fever	Initial symptoms and signs	CPR	cTn	ECG	CXR	Echo	MRI	EMB	IVIG/Steroid/Other	Anti-viral agents	ECMO	CVVH	Outcome
El-Assaad et al. (4)	10 Y	M	7 D	+	Fatigue, cough, diarrhea, vomiting, myalgia, nonpruritic rash	−	↑	ST/CHB	Pneumonia	EF32%	−	−	IVIG/MTP/Anakinra	Remdesivir	−	−	Survival
Gnecchi et al. (5)	16 Y	M	NA	+	Chest pain radiating to left arm	−	↑	STE	NA	EF52%	+	−	NA	Hydroxy-chloroquine	−	−	Survival
Fischer et al. (6)	15 Y	M	3 D	+	Persistent chest pain	−	↑	STE	NA	EF50%	+	−	NA	NA	−	−	Survival
Oberweis et al. (7)	8 Y	M	4 D	+	Cough, weight loss, fatigue	−	↑	STE	NA	FS21%	+	−	IVIG/Tocilizumab	NA	−	−	Survival
Giacomet et al. (8)	2 M	F	2 D	+	Non-bloody diarrhea, vomiting	−	↑	ST	Unremarkable	EF53%	−	−	IVIG	NA	−	−	Survival
Lara et al. (9)	2 Y	F	3 D	+	Fatigue, diffuse abdominal pain, nausea, vomiting, bradycardia	+	↑	CHB	Normal	EF27%	−	−	IVIG	NA	−	−	Survival
Kesici et al. (10)	2 Y	M	NA	NA	Nausea, vomiting, poor oral intake	+	↑	NA	Bilateral infiltration	EF15%	−	−	NA	NA	+	−	NA
									Cardiomegaly								
									Pleural effusion								
Kohli et al. (11)	15 Y	F	NA	+	Headache, vomiting, fatigue	−	↑	AFib	Minimal vascular engorgement	FS20%	−	−	IVIG/MTP/Anakinra	NA	−	−	Survival
Tseng et al. (12)	5 Y	M	1 D	−	Fatigue, vomiting	−	↑	mVT	Cardiomegaly	NA	−	−	IVIG/MTP	NA	+	−	Survival
									Pulmonary edema								
Nishioka et al. (13)	15 Y	M	1 D	+	Fatigue, abdominal pain	−	↑	AVB	Cardiomegaly	EF25%	−	−	DEX	Remdesivir	+	−	Survival
Buitrago et al. (14)	12 Y	F	2 D	+	Headache, neck pain, nausea, diarrhea, lethargy	+	↑	STE	Pulmonary edema	EF11%	−	+	MTP	NA	+	−	Survival
Phan et al. (15)	9 Y	M	4 D	+	Sore throat, dry cough, chest pain, fatigue, dyspnea	+	↑	STE	Cardiomegaly	EF30%	−	−	DEX	NA	+	+	Survival
									Pulmonary opacification								
This case (2022)	9 M	M	1 D	+	Poor activity, lip cyanosis, dyspnea	+	↑	ST	Cardiomegaly	EF19%	+	−	IVIG	Remdesivir	+	+	Survival
									Bilateral infiltration								

CPR, cardiopulmonary resuscitation; D, days; M, months old; NA, not available; Y, years old; AFib, atrial fibrillation; AVB, atrioventricular block; CHB, complete heart block; CVVH, continuous venovenous hemofiltration; cTn, cardiac troponin; DEX, dexamethasone; ECG, echocardiography; Echo, echocardiography; ECMO, extracorporeal membrane oxygenation; EF, ejection fraction; EMB, endomyocardial biopsy; FS, fractional shortening; IVIG, intravenous immunoglobulin; MRI, magnetic resonance imaging; MTP, methylprednisolone; mVT, monomorphic ventricular tachycardia; PSVT, paroxysmal supraventricular tachycardia; ST, sinus tachycardia; STE, ST elevation.

To the best of our knowledge, our patient was the youngest case of fulminant myocarditis due to COVID-19 successfully treated with ECMO as a bridge to a good outcome. Fulminant myocarditis is the most severe type of myocarditis and is predominantly caused by viral infections. It is characterized by a sudden and severe inflammation of the myocardium, resulting in cardiogenic shock, ventricular tachyarrhythmias, or bradyarrhythmias with the need for hemodynamic support (17). Direct viral infection, hypoxia-induced apoptosis, and association with the cytokine storm may be the causal mechanisms of arrhythmias (18).

The common indication for performing cardiac MRI in children is to identify myocardial injury and to detect inflammatory features to distinguish acute myocarditis from noninflammatory cardiomyopathies, which is quite different to adults distinguishing myocarditis from coronary ischemia (19). In a small study of 23 patients with clinically suspected myocarditis, increased T1, T2, and extracellular volume demonstrated specificities between 74% and 89% and sensitivities between 86% and 91%. 86% had late gadolinium enhancement and signal increases on T2-weighted imaging was present in 57% (20). Our patient's image was compatible with these findings, which showed the possible consistency within the infantile population.

Among treatment options, we managed possible cytokine storm with IVIG and CVVH. IVIGs are commonly used to treat inflammatory diseases with cardiac involvement due to their immune-modulating activity. CVVH may limit further cardiotoxicity from possible indirect damage in fulminant hypercytokinemia-associated SARS-CoV-2 (15). Remdesivir, a direct-acting nucleotide prodrug inhibitor of the SARS-CoV-2 RNA-dependent RNA polymerase, may shorten the recovery time in patients hospitalized with COVID-19 and was associated with fewer days of subsequent oxygen use in patients receiving oxygen and shorter subsequent duration of mechanical ventilation or ECMO (21). The U.S. Food and Drug Administration had expanded the approval of remdesivir use in April, 2022 for hospitalized pediatric patients with COVID-19 28 days of age and older and weighing at least 3 kg. Hence, our patient met the criteria for use of remdesivir. As for treatment options for cardiogenic shock, we selected VA-ECMO because of the rapid worsening of the patient's cardiac function. A diagnosis of cardiogenic shock carries a high risk of mortality, especially without definitive intervention. In the case series of twelve cases, ECMO was only applied in five cases. Early intervention with mechanical circulation support (MCS) can be lifesaving and ECMO is an attractive choice of MCS because it can be deployed emergently. The probability of a brisk cardiac recovery in myocarditis is also high compared with cardiomyopathy (19). The reason why ECMO was not chosen as a therapeutic option in the rest of the cases may possibly due to the variability of disease fulminancy and the availability of MCS. The specific role of ECMO support for pediatric COVID-19 patients with severe cardiac involvement is still evolving. Our case serves as an example of early initiation of VA-ECMO for cardiogenic shock in a COVID-19 case that was younger than 1 year of age.

Of note, our patient was initially managed at the emergency department with an impression of acute bronchiolitis due to wheezing breath sounds and dyspnea, which was a less common presentation compared to the series of twelve case reports. This suggests that thorough evaluations for all organ systems should be carefully performed in all COVID-19 patients so that myocardial involvement can be identified and differentiated from other respiratory complications, especially in those who are extremely young.

## Data Availability

The original contributions presented in the study are included in the article, further inquiries can be directed to the corresponding author.
